# A qualitative study of how inter‐professional teamwork influences perioperative nursing

**DOI:** 10.1002/nop2.422

**Published:** 2019-11-27

**Authors:** Thekla Holmes, Anne Vifladt, Randi Ballangrud

**Affiliations:** ^1^ Department of Health Sciences Gjøvik Faculty of Medicine and Health Sciences Norwegian University of Science and Technology Gjøvik Norway; ^2^ Department of Surgery Innlandet Hospital Trust Gjøvik Norway

**Keywords:** operating rooms, patient safety, perioperative nursing, TeamSTEPPS^®^, teamwork

## Abstract

**Aim:**

To explore Norwegian operating room nurses’ perceptions of how team skills in the inter‐professional operating room team influence perioperative nursing in relation to patient safety.

**Design:**

A qualitative, descriptive study based on interviews.

**Methods:**

Ten operating room nurses (*N* = 10) employed in four Norwegian hospitals were interviewed individually. A qualitative inductive content analysis was conducted. The study was reported adhering to the Consolidated Criteria for Reporting Qualitative Research Checklist.

**Results:**

Three generic categories, containing three subcategories each, were identified illuminate the operating room nurses’ perceptions. The operating room team's team skills influence on (a) the quality of perioperative nursing, about task performance, result for the patient and learning; (b) the progress of perioperative nursing, by keeping focus on the task, being prepared and task distribution and (c) the operating room nurses’ work environment in the operating room, including confidence, stress and energy use and irritation or job satisfaction.

## 1 INTRODUCTION

Operating room (OR) nurses take care of surgical patients in a complex environment and under pressure of time in quickly changing, stressful circumstances. In order to achieve the team's goal of successful surgery and avoid patient harm, OR nurses in the role of the scrub nurse and circulating nurse cooperate closely with the rest of the inter‐professional OR team (Ministry of Education & Research, 2005).

Teamwork can be described as the interaction of two or more professionals about behaviours, cognitions and attitudes, which contributes to reaching shared goals through interdependent performance (Weaver, Feitosa, Salas, Seddon, & Vozenilek, 2013). Good knowledge, skills and attitudes towards teamwork in all team members are crucial for the team's performance (Alonso & Dunleavy, 2013).

Since inter‐professional OR team members rarely work together for more than one day at a time, they need considerable team skills, in order to reach the goal of a good patient outcome (Anders, France, & Weinger, 2013). Research has shown that the professions represented in the OR, that is OR nurses, surgeons and their assistants, anaesthetists and anaesthesia nurses, often have different perceptions of OR teamwork (Bogdanovic, Perry, Guggenheim, & Manser, 2015; Gillespie, Gwinner, Chaboyer, & Fairweather, 2013; Wauben et al., 2011). The greatest gaps in perception occur between surgeons and nurses, with surgeons scoring teamwork, communication, leadership and respect higher than nurses (Carney, West, Neily, Mills, & Bagian, 2010). OR nurses perceived respect as being most important in teamwork, in the sense of appreciation, understanding and constructive communication (Kaldheim & Slettebø, 2016). They experienced preoperative briefings and debriefings with the whole team as inadequate and said that it is difficult for OR nurses to stop the procedure if necessary (Wauben et al., 2011). Nakarada‐Kordic et al. (2016) found discrepancies in OR team member's mental models in respect of responsibility for and order of tasks during surgery.

In recent years, the World Health Organization (WHO, 2017) has increased the focus on teamwork in inter‐professional healthcare teams in order to improve patient safety. Nevertheless, approximately 27% of all adverse events in patient care globally are associated with surgical procedures (WHO, 2017).

## 2 BACKGROUND

Many of the adverse events in the OR are associated with teamwork and are therefore considered to be preventable (de Vries, Ramrattan, Smorenburg, Gouma, & Boermeester, 2008). Teamwork problems like poor communication, misunderstandings and interruptions often occur together with a lower level of performance of non‐technical skills, so‐called team skills (Siu, Maran, & Paterson‐Brown, 2014). Non‐technical skills can be defined as “cognitive, social and personal resource skills that complement technical skills and contribute to safe and efficient task performance” (Flin, O'Connor, & Crichton, 2008). Systems to assess OR team members’ non‐technical skills (Fletcher et al., 2003; Mitchell et al., 2013) identified that poor non‐technical skills are associated with more delays and tension in the OR team (Lingard et al., 2004; Mitchell et al., 2011), more issues related to equipment, instruments and supplies (Fischer, Tubb, Brennan, Soderdahl, & Johnson, 2015), more intraoperative events and errors (Siu et al., 2014) and team members not speaking up or following procedures (Künzle, Kolbe, & Grote, 2010). For this reason, poor non‐technical skills can have a negative effect on patient safety, for instance increased probability of morbidity and mortality (Johnson & Kimsey, 2012; Mazzocco et al., 2009; Neily et al., 2010; Weaver et al., 2010).

The implementation of the WHO Surgical Safety Checklist (SSC) (World Alliance for Patient Safety, 2008) has reduced mortality, complication rates, length of stay and number of adverse events associated with surgery (Bergs et al., 2014; Weller & Boyd, 2014). It has also strengthened OR teamwork by improving the sharing of critical information, confidence and team familiarity (Russ et al., 2013). OR nurses reported that they feel more like a part of the team and that the checklist gives them the opportunity to raise questions about equipment and procedures, which results in strengthened awareness, preparedness and systemization (Høyland, Haugen, & Thomassen, 2014; Wæhle, Haugen, Søfteland, & Hjälmhult, 2012).

Team skills and team behaviour in the OR have also been improved by team training (Gillespie, Chaboyer, & Murray, 2010; Nurok, Lipsitz, Satwicz, Kelly, & Frankel, 2010; Rutherford, 2017; Weaver et al., 2010). All inter‐professional team members must be included in teaching, simulation and implementation of tools and strategies (Baker, Salas, Battles, & King, 2017). Research has shown that surgery‐related morbidity, mortality and the number of patient safety‐related intraoperative events were reduced significantly after team training (Forse, Bramble, & McQuillan, 2011; Gillespie et al., 2010; Johnson & Kimsey, 2012; Neily et al., 2010; Weld et al., 2016; Weller & Boyd, 2014). At the same time, OR efficiency increased and there were fewer delays (Shams et al., 2016).

Team Strategies and Tools to Enhance Performance and Patient Safety (TeamSTEPPS^®^) is an evidence‐based team training programme, developed by the US Department of Defense and the Agency for Healthcare Research and Quality (AHRQ, 2014). TeamSTEPPS^®^ has been implemented in many US health institutions since 2006 and is now the national standard framework for team training in the USA (Baker et al., 2017). Even though research has shown measurable positive effects of team training with TeamSTEPPS^®^ on patient outcome and efficiency (Forse et al., 2011; Shams et al., 2016), healthcare around the world has not yet widely implemented team training into clinical practice and education. The team skills that represent the core competencies of teamwork according to TeamSTEPPS^®^ are described as: (a) communication, which is the process of exchanging information clearly and accurately among team members in a structured way; (b) leadership, which maximizes the activities of team members through securing understanding, information sharing and providing resources; (c) situation monitoring, which involves continuous and active scanning and assessing of the situation to gain information and awareness and (d) mutual support, which includes anticipating team members’ needs and supporting them (AHRQ, 2014).

Previous research consists mainly of quantitative studies concentrating on the OR team as a whole, and there are relatively few qualitative studies investigating teamwork in the OR. As far as we can see, little attention has been paid specifically to OR nurses’ team skills and their perception of teamwork in the OR. OR nurses are important members of the OR team and possess unique experience. Therefore, the aim of this study was to explore Norwegian OR nurses’ perceptions of how team skills in the inter‐professional OR team influence perioperative nursing in relation to patient safety.

## 3 METHODS

This study has a qualitative, descriptive design (Polit & Beck, 2012) and is based on interviews. It was conducted in accordance with Kvale and Brinkmann (2015) steps in interview surveys in order to gain profound and general knowledge by collecting narrative data.

### 3.1 Setting and sample

Operating departments at three general hospitals and one university hospital in the south‐east of Norway participated. None of these operating departments had performed team training prior to this study. A convenience sample of 10 OR nurses was recruited. OR nurses are registered nurses who have completed a postgraduate specialist training, comprising 18 months of fulltime education, including almost 50% clinical training (Ministry of Education & Research, 2005). The participants were approached by means of an invitation letter that their managers distributed by e‐mail. This letter provided information about the researcher and the study.

The OR nurses were working in different surgical specialties. Inclusion criteria were that they had (a) Norwegian postgraduate OR nursing education, (b) at least 2 years’ experience as an OR nurse and (c) minimum 50% permanent employment. All criteria were met, except for one participant who had only one year of experience. Additional participant characteristics are shown in Table 1. All co‐workers of the first author were excluded. An eleventh interviewee chose to terminate the interview, and all information from this interview was deleted.

**Table 1:** Description of the participants

Characteristics	Category	OR nurses (*N* = 10)
Gender	Female	9
	Male	1
Age	<40 years	5
	41–50 years	3
	>51 years	2
Experience as OR nurse	<5 years	5
	6–10 years	2
	>11 years	3

Abbreviation: OR, operating room.

### 3.2 Data collection

Data collection took place in April and May 2017. The pilot tested, semi‐structured interview guide with pre‐determined, open‐ended questions (see Appendix S1) was based on the team skills described in the TeamSTEPPS^®^ framework: communication, situation monitoring, mutual support and leadership (AHRQ, 2014).

All individual interviews were conducted by the first author in a quiet location at the participant's workplace during working hours. The interviews lasted an average of 40 min (range 26–54 min) were audio‐recorded, and field notes were made.

The interviewer gave a short definition of each team skill based on TeamSTEPPS^®^, before asking how these skills in the OR nurse her‐/himself and the other team members affect their practice of OR nursing during the immediate preoperative, intraoperative and immediate postoperative phases of surgical procedures. All interviewees were asked the same questions, in addition to more detailed follow‐up questions on topics important to the aim of the study. Finally, each interviewee was asked to recall a case where they had experienced the teamwork in the OR as especially good or bad and they were able to add additional information or comments. The researcher had no more contact with the participants afterwards.

### 3.3 Data analysis

**Figure 1:**
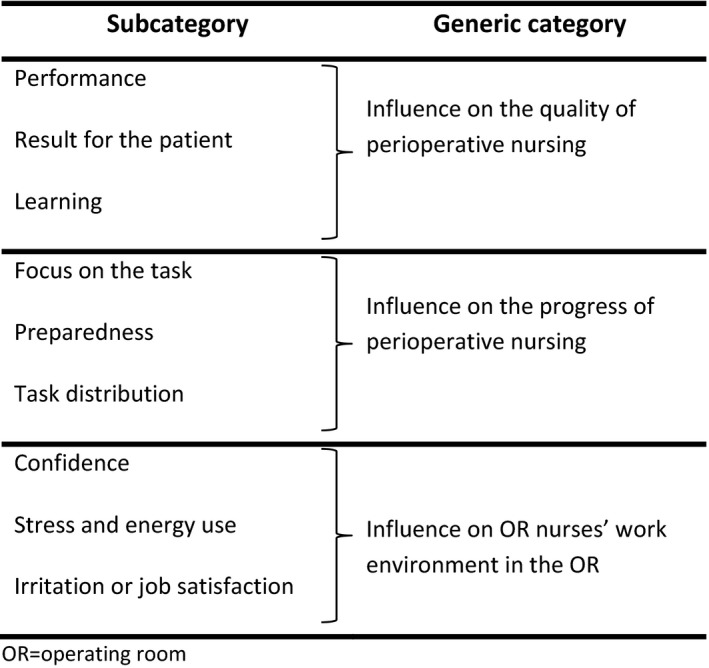
Operating room (OR) nurses’ perception of how the team skills of the OR team influence perioperative nursing in relation to patient safety

Following verbatim transcription of all interviews by the first author, an inductive content analysis in accordance with Elo and Kyngäs (2008) was performed, based on the manifest content of the data. During the preparation phase, each interview was defined as one unit of analysis. Familiarity with the texts and a general understanding of the content were achieved by reading the interviews repeatedly. In the organizing phase, all meaningful phrases were identified, coded openly by writing notes and headings in the text and condensed. Groups of condensed phrases that shared the same meaning were gathered into subcategories and similar subcategories were grouped together (Table 2). Through further abstraction, subcategories were grouped into higher‐order generic categories, which are internally homogeneous but mutually exclusive and describe all of the manifest content of the data material. Each of the three generic categories and nine subcategories that emerged was named using words characteristic of its content. The reporting phase involved making an overview (Figure 1) and giving a description of the results. The first author (TH) had main responsibility for the analysis with assistance from the co‐authors (AV and RB). All three authors participated in reporting the results. The results have been reported in accordance with the Consolidated Criteria for Reporting Qualitative Research Checklist (see Appendix S2) (Tong, Sainsbury, & Craig, 2007).

**Table 2:** Illustration of coding, categorizing and abstraction from text to subcategory

Transcribed interview text, identification of meaningful phrases	Coding sheet with condensed phrases	Codes	Subcategory
If I’m not able to communicate with them, we won't be able to do a good job (2.28). Then I might make a mistake (2.29) or might not perform OR nursing so well (2.30). If I ask for something I think I need and then it wasn't that one and then you have to run and get another thing (2.31), because I said something wrong, or you misunderstood me (2.32). (Participant 2)	2.28 Without communication cannot do good job	Performance	Performance
	2.29 Without communication can make a mistake	Mistake	
	2.30 Without communication not so good a performance	Performance	
	2.31 Poor communication can give wrong equipment	Equipment	
In positioning and change of position during surgery (…) you just have to take on that role, be clear, “Now we do this” (…) We are finished and it turned out the way I wanted (6.134). (Participant 6)	2.32 Poor communication can lead to misunderstanding	Misunderstanding	
	6.134 Good leadership gives right positioning	Positioning	
We help each other (…) we ask for advice (…) how to drape (9.56), how to position (9.57). (Participant 9)	9.56 Good support gives right draping	Draping	
	9.57 Good support gives right positioning	Positioning	

Abbreviation: OR, operating room.

### 3.4 Ethics

This study was planned and conducted according to the ethical guidelines for nursing research (Northern Nurses’ Federation, 2003). The study was approved by the Norwegian Social Science Data Services (NSD ref. 52820) and reported to the research departments of the participating hospitals. The ward management received information about the study by e‐mail and approved participation. All participants received written and oral information about the study and their right to withdraw from the study at any time, without giving any reason and without any negative consequences. Written consent was obtained prior to the interviews. Confidentiality was ensured by anonymizing all data material.

## 4 RESULTS

Three generic categories were identified from the data material and each of them consists of three subcategories as shown in Figure 1.

### 4.1 Influence on the quality of perioperative nursing

This generic category expresses the OR nurses’ perception that the performance of perioperative nursing is better when there are good team skills in the OR team than when the team skills are poor. This was expressed in three subcategories: performance, result for the patient and learning.

#### 4.1.1 Performance

The participants said that all four team skills are of importance to whether the OR nurses’ tasks are performed correctly and well, or not. They described being dependent on the rest of the team in their performance:

It affects my job. If they are not doing a good job, nor will I and vice versa. (Participant 2)

At the same time, OR nurses strive to do their tasks in a good way, even though this might be time‐consuming and tiring:

My standard is the same, independent of who the patient is, or who I’m working together with. But it’s easier to achieve this if communication is good. (Participant 6)

According to the OR nurses, good team skills, especially good communication, contribute to good quality in perioperative nursing. They explained this as doing things correctly, that you do not make mistakes or forget something and that mistakes and oversights are detected and corrected. This includes following procedures, performing the SSC and considering the patient's needs at all times.

The participants perceived that poor communication or situation monitoring, along with experiencing a lack of mutual support or leadership, can lead to intraoperative events such as faulty positioning or draping, lacking equipment, or forgetting a catheter or warming blanket.

The OR nurses perceived constructive criticism, willingness to learn, planning and a good tone as resulting in better performance of perioperative nursing, whilst unnecessary communication, noise, loss of concentration, stress, insecurity and irritation have a negative influence.

#### 4.1.2 Result for the patient

The OR nurses expressed their view of the importance of all four team skills in order to achieve a good result for the patient. They said that they can avoid patient harm, complications and negative consequences for the patient and ensure patient safety by being able to do a better job and avoiding intraoperative events. Throughout all interviews, focus on the patient's best was a common feature:

We are good at asking each other for help and support (…) so the operation goes as well as possible and the patient is not harmed. (Participant 6)

Good communication makes it easier for OR nurses to speak up about risks to patient safety. They perceived that adverse events such as hypothermia, injury due to positioning, infection and extensive blood loss might occur partly because of poor team skills. Even though the OR nurses emphasized that poor communication or support from others should not influence patient outcome, one commented:

If you aren’t good at leading the OR team, it can result in misunderstandings, which can have serious consequences, complications, for the patient. (Participant 3)

#### 4.1.3 Learning

Independent of experience, most participants highlighted a continuous improvement in the quality of perioperative nursing through learning. All four team skills were perceived as important for learning. Things that can be done better can be pinpointed through good communication and mutual support:

I want to learn from the mistakes I might make. (…) It is not about exposing someone, but about learning and becoming better. (Participant 7)

The participants perceived that learning from each other, gaining understanding for other team members and improving procedures and routines also improve quality and patient safety for future patients. Such learning and improvement are inhibited by poor team skills.

### 4.2 Influence on the progress of perioperative nursing

This generic category expresses the OR nurses’ perception that progress in their tasks and in the operation itself is better when the OR team displays good team skills than when team skills are poor. Progress was described as depending on focus on the task, preparedness and task distribution.

#### 4.2.1 Focus on the task

The participants perceived that being able to maintain undivided attention on their current task contributes to good progress. They thought that focus or concentration could be increased by good communication, for instance clear messages and constructive criticism. Good situation monitoring, mutual support and leadership enable the OR nurse to focus on her/his own tasks without being disturbed or interrupted. Poor communication, for example lack of information, inappropriate or unnecessary remarks, poor leadership and situation monitoring, or having to take on others’ tasks, can result in delays because they lose focus on their own task:

You lose some focus when you have to watch what is going on around you at the same time (…) so you might not be ready with the instruments when you should be. (Participant 1)

#### 4.2.2 Preparedness

Another way to avoid unnecessary waits and save time is according to the OR nurses being prepared and having things ready. Good team skills, especially communication by asking questions and planning, are in their opinion necessary in order to have instruments and equipment readily at hand, but also to be prepared for patients’ special needs.

Poor communication and situation monitoring were thought to be the team skills that could cause most delays and give rise to extra work:

Things could run a bit more smoothly, if you knew things beforehand. You could be prepared (…) but then maybe the surgeon has to wait while we get the equipment. (Participant 10)

#### 4.2.3 Task distribution

The operation's progress also depends on a good flow of tasks. Good flow meant to the OR nurses that everyone knows and performs their tasks and that time and resources are exploited to the full. They said that good team skills contribute to better flow, whilst poor team skills give poorer flow. They talked about the importance of distributing and adjusting tasks according to team members’ knowledge and experience to the extent possible, through good mutual support and leadership. Good situation monitoring also enables team members to overlap roles, for example in positioning the patient and avoiding unnecessary use of time:

In big operations it is good when the surgeon comes in early to see everything is okay. To help find equipment, hold up the leg for skin disinfection … and at least offer to help. (Participant 4)

Good team skills were perceived to contribute to an early start of the operation and enable the team to manage a big programme, by compensating for unexpected delays or inexperienced team members to some degree:

Even if things happen during the operation, it won’t tip the whole load, but you can make up for it and still make it work. (Participant 4)

The participants also stressed that a mutual understanding of what will happen during the operation and knowing the proper order of instruments to be used, is important for progress and can be achieved through good team skills such as good communication and mutual support.

### 4.3 Influence on OR nurses’ work environment in the OR

This generic category expresses the OR nurses’ perception that important aspects of their work environment in the OR, including confidence, stress and energy use and irritation or job satisfaction, are affected by the OR team's team skills. The participants described how their experience of the work environment can affect the quality and progress and in consequence patient safety in perioperative nursing.

#### 4.3.1 Confidence

The participants perceived that good team skills create a sense of security and calmness, especially in complex situations:

If there is something the other person doesn’t know how to do, maybe I can take control. (…) That feels safe for the others and for me. (Participant 9)

Good team skills such as asking for help or looking out for each other were thought to inspire mutual trust and a sense of achievement, satisfaction and confidence.

Poor team skills such as poor communication, including unclear messages or being yelled at, were perceived to create insecurity and a feeling of being inadequate. The same was said of having too much responsibility or lacking support and when other team members are not concentrated on their tasks and the situation. The OR nurses stated that these feelings can have a negative influence on their performance, especially when they are inexperienced:

The whole situation will be influenced a little by this insecurity (…) which can eventually be detrimental to patient safety. (Participant 8)

#### 4.3.2 Stress and energy use

Poor team skills such as misunderstandings, interruptions and not being able to trust others to do their job properly can create stress, which again can increase the risk of making mistakes:

I get all tensed up and then I (…) give the wrong instrument. (Participant 5)

The participants explained how they have to use energy in order to prevent stress, irritation or insecurity from affecting their performance, which makes work tiring.

Good team skills, on the other hand, are perceived to reduce or prevent stress, making their job both physically and mentally easier:

We have a lot of heavy lifting and if I work with an anesthesia nurse who helps me remove the ends of the operating table, for example (…) without saying a word, to get the patient into lithotomy position in a quick and smooth way,(…) that is very positive for me. (Participant 7)

Planning, appropriate role distribution and receiving help without asking were mentioned as examples of stress‐reducing behaviour. Good team skills also made the OR nurses feel equal and strengthened teamwork by making them supportive towards the rest of the team.

#### 4.3.3 Irritation or job satisfaction

Behaviour connected to poor team skills such as unnecessary talk, inappropriate comments, lack of focus or others not taking responsibility for joint tasks can cause irritation and a bad atmosphere. Good team skills, especially good communication, were said to reduce irritation and help prevent conflicts.

Good team skills, including positive feedback and willingness to learn, were perceived to create good interaction and mutual respect. The OR nurses connected good teamwork with a good atmosphere and well‐being. However, the participants said clearly that irritation and a bad atmosphere should not affect the patient:

I don’t do a worse job (…) if the atmosphere isn’t so good. (…) But it’s more fun being there. (Participant 9)

## 5 DISCUSSION

The results show that the team skills in the inter‐professional OR team influence the quality and the progress of perioperative nursing and the work environment in the OR. The complexity of tasks and different patient requirements in the OR demand a high level of interdependent teamwork in order to meet patient needs and avoid patient harm. According to the OR nurses, good performance of perioperative nursing, including performing the SSC, is highly dependent on good team skills in the OR team. Even though the SSC has improved patient safety and OR teamwork, it is often performed incompletely (Wæhle et al., 2012) and OR nurses experience a lack of preoperative briefings with the entire OR team, where they can get valuable information and discuss concerns (Wauben et al., 2011). Inter‐professional training initiated by ward management can improve team skills and make the SSC a common concern for all OR team members (Wæhle et al., 2012), which will enable OR nurses to ensure quality and patient safety as they are expected.

Further our findings demonstrate that good team skills in the OR team can enable OR nurses to contribute to avoiding negative consequences for patients undergoing surgery. Previous studies have found that patients’ risk of experiencing complications or death increase when team skills are poor and that communication failures, in particular, pose a threat to patient safety by causing intraoperative events (Lingard et al., 2004; Mazzocco et al., 2009). The OR nurses mentioned that it is easier to speak up about mistakes, oversights, risks and deviations from procedures when the OR team's team skills are good. The traditional hierarchy between nurses and surgeons can inhibit speaking up (Carney et al., 2010), while being respected as equal team members is important for OR nurses (Kaldheim & Slettebø, 2016). Improving the OR team's team skills through team training, including encouraging OR nurses to speak up, has been shown to reduce mortality and morbidity related to surgical procedures significantly (Forse et al., 2011; Johnson & Kimsey, 2012; Weller & Boyd, 2014).

Learning and improvement are indicators for patient safety culture (Sammer, Lykens, Singh, Mains, & Lackan, 2010). Our findings indicate that good team skills contribute to learning, sharing experience and tacit knowledge, and talking openly about mistakes. Good team skills help OR nurses to take responsibility and being concerned about a safe framework for perioperative nursing, such as continually improving procedures. Even though the OR nurses emphasized the importance of team skills on their performance and patient safety without hesitation, they seemed to have difficulties in drawing direct lines between team skills and concrete tasks. Since tacit knowledge is unconscious or implicit, OR nurses need to become aware of these connections through training and reflection in education and as a team in the OR.

The OR nurses perceived that good team skills in the OR team enable them to maintain focus on their current task and complete it quickly, while poorer team skills, causing disturbances and interruptions, can lead to delays. Disrespectful communication could be one possible factor that disturbs OR nurses’ concentration (Kaldheim & Slettebø, 2016). According to the Systems Engineering Initiative for Patient Safety (SEIPS) model, there is a very complex interaction between technology, tools, organizational circumstances, tasks and surroundings, which influences work processes and practitioners (Holden et al., 2013). How OR nurses respond to these influences is partly individual and more qualitative evidence is needed to understand how good team skills help OR nurses to stay focused and reduce the use of time in the OR.

The OR nurse's preparedness was also said to influence progress in perioperative nursing and the operating time by preventing delays in preparing the patient for surgery and waiting time during surgery. Information about the plan for the surgery, required positioning and equipment, was mentioned as assisting OR nurses to have things ready before the patient arrives at the OR. Previous observations have shown that many communication failures in the OR team result in delays and inefficiency (Lingard et al., 2004). OR nurses need to be able to anticipate what might happen during surgery and be prepared in advance in order to save time and ensure patient safety (Høyland et al., 2014). It is easier for experienced nurses to anticipate needs and act efficiently compared with those who have less experience (Lingard et al., 2004), which increases the need for good team skills in the OR team. Weld et al. (2016) found that team training was associated with shorter operating times and an improvement of on‐time starts.

The OR nurses perceived that good team skills facilitate the distribution of tasks in a way that makes the OR team as effective as possible. Knowledge of each other's responsibilities, strengths and weaknesses and sharing a common goal are necessary to work effectively as a team (AHRQ, 2014). Respectful team communication and adaptability between different professional identities in the OR team can increase OR efficiency (Kaldheim & Slettebø, 2016; Weller & Boyd, 2014). Thus, the OR team can compensate for unexpected delays that may arise, for example in the case of patients with challenging anatomy (Rutherford, 2017). This study also revealed that OR nurses are reluctant to lead the OR team, regardless of how long experience they have and even in situations where they have most knowledge, for example positioning the patient. Good leadership is a key skill in all teamwork (AHRQ, 2014) and we, therefore, encourage team training as part of OR nurses’ education and in all surgical wards (Weller & Boyd, 2014).

Further, the OR nurses experienced that good team skills can contribute to a good work environment where they can feel calm and confident, while inadequate team skills were connected to a poor work environment characterized by insecurity, stress, unnecessary use of energy and irritation. Previous studies have found that inadequate communication is often the reason for a bad atmosphere in the OR (Mazzocco et al., 2009). This can contribute to a wrong perception of each other's motives (Wauben et al., 2011), at the same time as tensions and stress spread easily among team members (Lingard et al., 2004). A good work environment, on the other hand, is associated with better performance and patient safety (Mitchell et al., 2011; West, Guthrie, Dawson, Borrill, & Carter, 2006) and can help the OR team adapt more easily to critical situations (Bogdanovic et al., 2015). The OR nurses explained the self‐enhancing mechanism of a good work environment, where team members are more likely to help each other when they themselves receive help when needed. Constructive criticism, appreciation and sympathy are expressions of good team skills that can increase OR nurses’ feeling of being respected and contribute to a good work environment (Kaldheim & Slettebø, 2016).

Our findings revealed a discrepancy in OR nurses’ perception of how much influence the work environment in the OR has on their performance. On the one hand, they agreed on the negative effects caused by problem behaviour and poor team skills, such as spending more time, making mistakes more easily or being un‐concentrated. On the other hand, they believed that by being professional and more experienced they could prevent a poor work environment from having a negative effect on their performance and patient safety. Professionalism does not include accepting disruptive behaviour and all professions must adhere to suitable professional behaviour in order to enhance the OR team's performance (Joint Commission, 2008). Team training by itself is not enough to create a good patient safety culture in the OR, unless all team members are committed to putting successful teamwork before their own individual performance (Weller & Boyd, 2014).

### 5.1 Limitations

The trustworthiness of the results, including credibility, dependability and transferability, was assessed throughout the process. The results are considered a valid description of OR nurses’ perceptions. However, the results might be influenced by sampling. Except for the inclusion and exclusion criteria and the information letter, the researcher did not have any influence on which participants were chosen by the ward leader. Furthermore, a thorough review of literature prior to the interviews and the first author's background as an OR nurse facilitated understanding of the data material, but misinterpretations of participants’ statements cannot be ruled out. The sample's gender distribution, with only one male OR nurse participating, reflects the workforce situation in Norway. The data from the male participant's interview did not differ from the rest.

## 6 CONCLUSION

The results of the study demonstrate that the team skills of the entire inter‐professional OR team have an impact on the performance of perioperative nursing and patient safety. Better team skills, which can be achieved by inter‐professional team training, can contribute to better quality and progress of perioperative nursing and a better work environment in the OR. Inter‐professional OR teams may promote patient safety by communicating about the connections between team skills, quality of work and work environment. Further research should explore intraoperative events in perioperative nursing and their association with team skills.

## CONFLICT OF INTEREST

All authors declare no conflict of interest.

## AUTHOR CONTRIBUTIONS

TH, AV and RB were responsible for the conception and study design. TH performed the data collection. TH, AV and RB contributed to the analysis of the data. TH, AV and RB were involved in drafting the manuscript and revising it critically for important intellectual content. All authors have read and approved the final manuscript.
