# Supplementary material for: A qualitative study of how inter‐professional teamwork influences perioperative nursing
Source: Nurs Open. 2019 Nov 27;7(2):571–80. doi: 10.1002/nop2.422 (PMC7024613; doi:10.1002/nop2.422)
Supplement: Supplementary file 1 [file NOP2-7-571-s001.docx]

Appendix 1: **INTERVIEW GUIDE**

**Information about the terms “teamwork” and “team skills” before the interview starts**

This is about *teamwork* = the interaction of two or more professionals regarding behaviours, cognitions and attitudes, which contributes to reaching shared goals through interdependent performance. Think about the OR team: OR nurses in the role of scrub nurse and circulating nurse, anaesthesia nurse, anaesthesiologist, surgeon, assistent and if relevant gastrolab, midwife etc. Not the ward staff, cleaning staff etc. The OR team’s *shared goal* is to perform successful surgery with a good result for the patient in an effectiv and secure way, attend to patient needs and avoid unnecessary harm, for example infection, injury caused by positioning, hypothermia, complications, forgotten sponges/instruments.

*Team skills:*

The thematic area is very comprehensive, so I will guide you with the questions. We will talk about four team skills: communication, situation monitoring, mutual support and leadership. However, these are closely connected to each other, especially communication, which is the lifeline in all teamwork. It doesn’t matter if we jump a little.

For each new team skill, I will give a short explanation of what it means. And just ask if you want me to read it again. Try to relax. This is supposed to be a conversation and there are no correct or incorrect answers. I want to hear about your everyday experiences, not something from a book. I want to explore your tacit knowledge about what influences your work, especially concerning patient safety.

We’ll stay inside the OR, concentrating on the roles of both the scrub nurse and the circulating nurse assigned to the specific OR team, not OR nurses serving several different ORs, participation in the trauma team etc. It might be easier if you think about a specific operation you normally perform, giving me some examples if possible.

Do you have any questions?

**Start of the interview**

1. **Communication** is the lifeline in all teamwork. It includes verbal and non-verbal exchange of information, feedback or response, ideas or feelings. The information must be conveyed clearly and precisely, accurately, relevantly and at the appropriate time.

*If you think about the other OR team members’ skills in communicating with you – how do they influence your performance of perioperative nursing?*

*How does it influence your performance of perioperative nursing if the others in the OR team have good or poor communication between themselves?*

*How do your own skills in communicating with the rest of the OR team influence your performance of perioperative nursing?*

1. **Situation monitoring** is a process of actively scanning and assessing the situation, i.e. how you are feeling and working, the patient and patient safety, the other team members, surroundings and equipment and the progression towards the goal. By being attentive, you can gain an understanding of the whole situation; you can detect changes, prevent or correct mistakes and be one step ahead.

*How do your skills in monitoring and assessing what is happening in the OR influence your performance of perioperative nursing?*

*How does it influence your performance of perioperative nursing if the others in the OR team are good or not so good at monitoring and assessing the situation in the OR?*

1. **Mutual support** is also called backup behaviour and is based on situation monitoring. Mutual support means assisting other team members to do their patient-related tasks if they can’t manage themselves. Mutual support is based on knowing each other’s roles and being willing to both ask for assistance and give assistance when needed. Mutual support can include practical help, reallocating workloads, giving feedback, speaking up for the patient or resolving disagreements.

*How does it influence your performance of perioperative nursing if the others in the OR team are good or not so good at supporting you?*

*How does it influence your performance of perioperative nursing if the others in the OR team are good or not so good at supporting each other?*

*How do your skills in asking for assistance and supporting the other team members influence your performance of perioperative nursing?*

1. **Leadership** ensures that the team stays focused and reaches its common goal of patient safety and high-quality patient care. The surgeon is the natural team leader, but in certain situations, the team member who is best qualified to address the problem may assume the leadership role. Leadership involves planning, delegating tasks and leading and coordinating activities. The leader must communicate this to the team, motivate and create a positive teamwork climate by being a good model and enhancing appropriate behaviour in the other team members.

*How do your skills in leading the others in the OR team influence your performance of perioperative nursing?*

*How does it influence your performance of perioperative nursing if the others in the OR team are good or not so good at leading you and the rest of the OR team?*

1. **Additional questions:**

*Can you tell me about a time when you experienced that you or the whole team did a very good or a very bad job concerning teamwork?*

*Is there anything else you would like to say?*

**Information in the end:**

All information will be handled confidentially. The interview will be transcribed and anonymized, then analysed together with the other interviews to find an answer to the research question. Your ward will get access to the results. It will not be possible to identify you or your hospital.

How did you experience the interview? Do you have any questions?

Thank you very much!
